# Sources of Food Affect Dietary Adequacy of Inuit Women of Childbearing Age in Arctic Canada

**DOI:** 10.3329/jhpn.v29i5.8899

**Published:** 2011-10

**Authors:** Sara E. Schaefer, Eva Erber, Janel P. Trzaskos, Cindy Roache, Geraldine Osborne, Sangita Sharma

**Affiliations:** ^1^Nutrition Research Institute, University of North Carolina at Chapel Hill, Kannapolis, NC 28081, USA; ^2^Department of Health and Social Services, Government of Nunavut, Nunavut, Canada; ^3^Department of Medicine, University of Alberta, Edmonton, Canada

**Keywords:** Arctic, Childbearing age, Cross-sectional studies, Diet, Dietary adequacy, Food consumption, Inuit, Maternal nutrition, Nutritional status, Canada

## Abstract

Dietary transition in the Arctic is associated with decreased quality of diet, which is of particular concern for women of childbearing age due to the potential impact of maternal nutrition status on the next generation. The study assessed dietary intake and adequacy among Inuit women of childbearing age living in three communities in Nunavut, Canada. A culturally-appropriate quantitative food-frequency questionnaire was administered to 106 Inuit women aged 19-44 years. Sources of key foods, energy and nutrient intakes were determined; dietary adequacy was determined by comparing nutrient intakes with recommendations. The prevalence of overweight/obesity was >70%, and many consumed inadequate dietary fibre, folate, calcium, potassium, magnesium, and vitamin A, D, E, and K. Non-nutrient-dense foods were primary sources of fat, carbohydrate and sugar intakes and contributed >30% of energy. Traditional foods accounted for 21% of energy and >50% of protein and iron intakes. Strategies to improve weight status and nutrient intake are needed among Inuit women in this important life stage.

## INTRODUCTION

Similar to many aboriginal populations worldwide, Inuit women of northern Canada have been undergoing a nutrition transition (1-4). Traditionally, these populations consumed a subsistence diet consisting of hunted and gathered foods, i.e. sea and land mammals, fish, shellfish, birds, and plants. However, within the last 50 years, consumption of imported foods high in fat and sugar and of relatively low nutritional quality has become common (5-11). Although the health benefits of the traditional diet are not well-understood, it has been recognized as nutrient-rich and protective against various chronic diseases (12-14). Studies of various aboriginal Arctic populations have shown that the recent dietary shift has resulted in decreased quality of diet characterized by excessive macronutrient intake (carbohydrate and fat) and insufficient vitamin and mineral intakes (15-19).

In Nunavut, an Inuit self-governed territory of northern Canada, the prevalence of obesity has increased approximately 88% more rapidly than the Canadian national average in the last 10 years. According to the Public Health Agency of Canada, approximately 58% of the Nunavut population was classified as overweight or obese (20). A recent study in three remote Nunavut communities found that 72% of Inuit adults were overweight or obese (21) and that nutrient-poor foods high in fat and sugar were prominent sources of dietary intake (11,22,23). Further, low intakes of many important nutrients, such as dietary fibre, calcium, folate, and vitamin A, D, and E, were prevalent (22).

Poor nutrition and related conditions, such as obesity, can threaten the quality of life for all population groups but they are of particular concern for women of childbearing age due to increased risks to reproductive health. Obesity is associated with reduced fertility, and both obesity and diabetes are associated with an increased risk of pregnancy-related complications that can harm the mother and the offspring (24-34). In addition to the reproductive risks associated with these symptoms of overnutrition, it is well-established that certain micronutrient deficiencies also carry risks for reproductive outcomes. For example, folate deficiency in the first three weeks of pregnancy is a leading cause of infant neural tube defects, and vitamin A deficiency has been suggested to be associated with congenital heart malformations (35-38). Both maternal obesity and micronutrient deficiency are predictors of the nutrition status of an offspring and development of chronic disease in adulthood (39-41).

Data are lacking on how the nutrition transition has specifically affected the diet of women of childbearing age in Nunavut and the potential implications on reproductive health. However, results of a study of Inuit in Arctic Quebec and Baffin Island revealed that birth defects were higher than in the general population, and vitamin A and folate deficiencies were cited as possible contributors to these high rates (29). Due to the potential impacts of poor maternal nutrition on pregnancy outcomes and the health of future generations, specific examination of the current diet of women of childbearing age is warranted in this region of transitioning diet. The objectives of the present study were to examine nutrient intake, dietary adequacy, and sources of main foods contributing to energy and selected nutrient intakes among Inuit women of childbearing age in Nunavut. Such data are necessary to inform the development of an intervention programme to improve dietary adequacy in this high-risk population.

## MATERIALS AND METHODS

### Setting

This cross-sectional study was conducted among three remote communities in the Kitikmeot region (Fig.) of Nunavut, the easternmost of three territories in Arctic Canada. Of a population of approximately 30,000, ~85% self-identify as Inuit. Inuktitut and Inuinnaqtun are the local languages. With a median age of 23 years, Nunavut has the youngest population of any province or territory in Canada (42).

**Fig.**
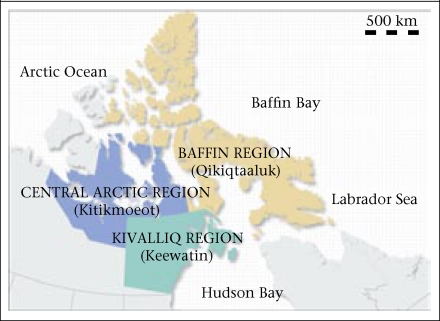
Map of Nunavut's three regions (43)

The three participating communities were identified by the Government of Nunavut that represented varying levels of the Inuit population and socioeconomic status. Community populations have been previously described (44) but, in brief, range from 800 to 1,500 people, 80%-90% of whom self-identify as Inuit. Their median age ranges from 20 to 26 years and employment from 40% to 60% (42). One community is a regional centre with a larger non-aboriginal population and greater engagement in the wage economy than the other two. Like many Nunavut communities, each has two food stores that obtain food primarily through shipments from the south via airplane year-round and also via barge/sealift once per year when the ice melts. Because of high cost of transportation and storage, prices of imported foods tend to be elevated compared to prices in southern Canada (45). Food is also obtained, to varying degrees, by traditional means, e.g. hunting and fishing.

### Collection of data

Data were collected as part of a larger study to assess the adequacy of diet among adult Inuit living in the Nunavut communities, and the study protocol has been previously described (44). Briefly, participants were recruited through random selection using up-to-date community housing maps provided by the local government. This method ensured sampling from areas with varied proximities to food stores and land resources. One resident per household was recruited, excluding residents aged less than 19 years (children) and pregnant/lactating women due to their different nutritional requirements and possible changes in dietary habits. Response rates in the three communities ranged from 69% to 93%. The present study reports data from women of childbearing age (19-44 years) included in the assessment.

Dietary data were collected during June–October 2008 using an interviewer-administered, culturally-appropriate quantitative food-frequency questionnaire (QFFQ) developed specifically for Inuit in Nunavut (23). The QFFQ was designed using single 24-hour recalls (n=87) to capture all foods/beverages typically consumed, including traditional foods not available in stores and those available seasonally. The QFFQ included 150 food items consumed throughout the year (12 breads and cereals; 65 meat, fish, and poultry; 12 dairy; one non-dairy creamer product; 13 fruits; 19 vegetables; 14 desserts and snacks; nine beverages; two sugar and sweetener products; three alcoholic drinks), 39 of which were traditional foods. The QFFQ has been previously validated using 24-hour recalls collected in the same population, and the two methods showed relative agreement of 83% for energy, 94% for sugar, 81% for macronutrients, and 84% for micronutrients, indicating that the QFFQ was a valid tool to assess dietary adequacy in this population (46). A population-specific food-composition database was developed using data from the Canadian food-composition tables within NutriBase, Clinical Nutrition Manager (version 7.17) (CyberSoft Inc., Phoenix, AZ), supplemented with data from the Canadian nutrient file.

Heights and weights of the participants were measured in duplicate and recorded on an additional anthropometry form. Heights were recorded to the nearest centimetre using a stadiometer, and weights were recorded to the nearest 1/10 of a kg using a digital scale. Before measurement, the participants were asked to remove shoes and heavy outer clothing, such as jackets. Weight was adjusted for clothing: one kg for light clothing, 1.5 kg for medium-weight clothing, and two kg for heavy clothing. If the participant declined to be measured, self-reported measurements were recorded. Two percent of the participants refused to have their heights and weights measured and self-reported the values instead.

Data were collected by community staff trained by the Principal Investigator in the administration and anthropometric measurements of the QFFQ. For participants whose primary language was not English, either an interviewer fluent in the local language conducted the survey or a local interpreter was used.

### Analysis of data

Data were analyzed using the SAS statistical software (version 9.1) (SAS Institute, Inc., Cary, NC). The mean, standard deviation (SD), and median daily energy and selected nutrient intakes were calculated from food intakes (excluding supplements which were taken by 12% of the population). Participants who reported extreme energy intake (<2,092 kJ (500 kcal) or >29,288 kJ (7,000 kcal); n=3) were excluded from the analysis. Dietary adequacy was determined using the estimated average requirements (EAR) for women per dietary reference intake (DRI) age-groups (19-30 years and 31-50 years) (47). If the EAR was not available, as for dietary fibre, vitamin D, vitamin K, pantothenic acid, potassium, sodium, and calcium, the adequate intake (AI) was used instead. The contribution of key food items to energy, macronutrients, sugar, fibre, calcium, iron, folate, and vitamin D intake was also determined.

### Ethical aspects

The approval of the Institutional Review Board was obtained from the Committee on Human Studies at the University of Hawaii and the Office of Human Research Ethics at the University of North Carolina at Chapel Hill. The Nunavut Research Institute licensed this study. The participants were remunerated for their time with C$ 25 gift cards for local stores. A signed consent form (available in English and the local languages) was obtained from each study participant

## RESULTS

The participants included 106 women with a mean±standard deviation (SD) age of 34.1±6.6 years and a mean body mass index (BMI) of 29.7±7.9 kg/m^2^ (Table 1).

**Table 1.** Demographics of Inuit women aged 19-44 years (n=106)

Demographics	Mean (SD)	
Age	34.1 (6.6)	
BMI (kg/m^2^)*	29.7 (7.9)	
	Women	
	No.	%
BMI (kg/m^2^)*		
<25.0 (normal)	28	28.3
25.0-29.9 (overweight)	31	31.3
≥30.0 (obese)	40	40.4
Supplement use		
Takes no supplement	93	88.6
Takes some form of supplement	12	11.4
Multivitamins	4	33.3†
Iron	3	25.0†
Calcium and/or vitamin D	3	25.0†
Prenatal	1	8.3†
Vitamin A	1	8.3†

* BMI data not available for six participants;

† Of the 12 individuals who reported supplement use;

BMI=Body mass index;

SD=Standard deviation

Almost 72% of the participants were overweight or obese according to the World Health Organization (WHO) classifications (48). Only 11.4% of the women reported taking a micronutrient supplement. Table 2 presents the mean, SD, and median energy and selected nutrient intakes, along with available nutrient DRIs for reference purposes. The mean daily energy intake of the study population was 13,188 kJ (3,152 kcal), which exceeded the minimum recommended energy intake of 7,531 kJ (1,800 kcal) (48) while the mean dietary fibre (15 g), potassium (4 g), vitamin D (5 μg) and vitamin E (4 mg) fell below the recommended intakes.

Table 3 shows that when intakes of selected nutrients were compared with their corresponding EARs, most (99%) women fell below the recommended level for vitamin E. More than 50% were below the EAR for potassium, >25% for folate, and >15% for magnesium and vitamin A. More than 90% of the women consumed dietary fibre below the AI while >50% were below the AI for vitamin D and potassium. More than one-third of the women were below the AI for calcium and vitamin K. Iron, niacin, riboflavin, thiamine, pantothenic acid, vitamin C, vitamin B-12, vitamin B-6, selenium, sodium, and zinc were consumed below the recommended levels by less than 15% of the women (data not shown).

The top 10 food-groups contributing to energy, macronutrients, sugar and fibre intakes (Table 4), and selected micronutrient intakes are also presented in Table 5. Foods classified as non-nutrient-dense (store-bought foods, particularly foods of high-energy density that are micronutrient poor) contributed the most energy (31%), fat (22%), carbohydrate (51%) and sugar (76%) intakes. Such foods, i.e. soft drinks, potato chips, cookies, and non-dairy coffee whitener, are those with high in fat/energy and/or relatively low-nutrient content and are not traditional foods, or classified in other food-groups, such as fruits/vegetables or grains (23). Traditional land foods, i.e. meat, organs, and fat from caribou, polar bear and musk ox, and sea foods, i.e. seal, muktuk (whale skin and fat), and locally-caught fish, accounted for 21% of energy intake and more than 50% of protein and iron intakes. White bread was the largest contributor to folate intake (31%) and was a significant contributor to calcium intake (24%) as well. However, dairy foods accounted for the greatest proportion (31%) of calcium intake.

## DISCUSSION

This is the first study that has examined dietary intake of Inuit women of childbearing age in Nunavut, and the results highlight various concerns regarding the diet of this population. Overweight and obesity were highly prevalent, and a number of nutrients, including dietary fibre, potassium, vitamin D and E, were consumed by more than half of the women below levels deemed to be adequate. Folate, calcium, magnesium, and vitamin A and K were also inadequately consumed among many women but to a lesser extent (16-46%).

A woman's pre-conception nutritional intake is a predictor for poor nutrition during pregnancy (49), which can have lasting effects on the health of the offspring. The foetal programming hypothesis proposes that environmental stimuli during intrauterine development may cause epigenetic alterations that permanently alter the functioning of the organism (50,51), and the intrauterine environment is associated with maternal diet and nutrient supply (52). Epidemiological studies provide evidence that maternal undernutrition and conditions relating to overnutrition, i.e. obesity and diabetes, are associated with an offspring's increased risk of developing chronic disease in adulthood (53,54).

There is also evidence that micronutrient deficiencies, which may be caused by insufficient and/or inappropriate dietary intake, can influence pregnancy and also metabolism of the offspring. For example, vitamin D status during pregnancy has been shown to affect preterm delivery, gestational diabetes, and also intrauterine skeletal mineralization (55-57). Vitamin D inadequacy as seen in the present study population highlights an area of concern for Inuit women, especially since adequate vitamin D from food sources is crucial for inhabitants of the northern regions who have very limited sunlight exposure, particularly during winter months (58).

Some intrauterine micronutrient deficiencies may lead to severe developmental abnormalities or congenital malformations, which have been documented as higher among aboriginal populations compared to non-aboriginal populations in Canada (29,59). For example, the folate status of a woman at conception may influence the development of neural tube defects (NTDs) (36). To increase folate intake among women of childbearing age, folic acid fortification of wheat has effectively reduced the incidence of NTDs in Canada by approximately 50% (60). In the present study, white bread (which included fortified products) was the food source accounting for the largest portion of folate intake. Despite this, the high prevalence of folate inadequacy among the study population may signify a need for further efforts to improve folate intake among Inuit women of childbearing age via foods rich in folate, supplementation and/or increased consumption of fortified foods. In Canada, NTDs also remain a risk among obese women, even subsequent to folic acid fortification of wheat flour (35), which may be of consequence in the Inuit population given the high rates of obesity.

**Table 2.** Energy and selected nutrient intake of Inuit women aged 19-44 years (n=106)

Nutrient	Mean	SD	Median	DRI*†
Energy (kJ)†††	13,188	5,151	12,609	7,531‡
Carbohydrate (g)	381.7	172.2	361.4	NA
Sugar (g)	195.2	126.7	176.0	<25% of energy
Fat (g)	98.0	38.7	93.2	NA
Monounsaturated fat (g)	33.3	13.6	30.8	NA
Polyunsaturated fat (g)	14.7	6.0	14.3	NA
Omega-3 (g)	1.7	1.4	1.2	NA
Omega-6 (g)	10.9	4.6	10.8	NA
Cholesterol (mg)	510.9	378.1	447.0	As low as possible
Protein (g)	176.2	103.3	154.0	NA
% of energy from CHO	48.5	9.2	48.3	45-65¶
% of energy from fat	28.3	5.1	28.5	20-35¶
% of energy from protein	21.8	7.3	21.4	10-35¶
Fibre (g)	15.2	6.8	14.1	25§
Calcium (mg)	1,261.6	627.3	1,143.5	800§
Folate**(μg)	418.2	182.6	398.6	320††
Iron (mg)	30.8	18.7	26.9	8.1††
Magnesium (mg)	401.1	164.5	378.0	265††
Niacin (mg)	37.4	19.0	32.6	14††
Pantothenic acid (mg)	10.7	6.2	9.5	5§
Potassium (g)	4.4	1.8	4.1	4.7§
Riboflavin (mg)	4.3	2.2	3.9	0.9††
Saturated fat (g)	34.9	13.9	33.5	<10% of energy‡‡
Selenium (μg)	183.3	222.1	143.3	45††
Sodium (g)	4.3	2.0	3.9	1.5§
Thiamine (mg)	2.4	1.1	2.2	0.9††
Vitamin A (RAE¶¶) (μg)	1,016.4	859.8	808.7	500††
Vitamin B-12 (μg)	16.9	15.3	12.9	2.0††
Vitamin B-6 (mg)	2.2	1.0	2.0	1.1††
Vitamin C (mg)	208.3	132.8	182.3	60††
Vitamin D (μg)§§	5.2	5.0	3.7	10§
Vitamin E (mg)***	4.3	2.2	4.0	12††
Vitamin K (μg)	129.5	116.3	95.2	90§
Zinc (mg)	22.6	14.6	19.9	6.8††

* The DRIs are presented in this table as a reference, using adequate intake, estimated average requirement for women aged 19-50 years. Acceptable macronutrient distribution ranges, and recommendation on saturated fat intake by Joint WHO/FAO (Institute of Medicine of the National Academies, 2005 and Joint WHO/FAO Expert Consultation, 2003);

† Value for ages 31-50 years chosen as population studied had a higher percentage of women in this age-category;

‡ Estimated amount of energy needed to maintain energy balance for women aged 31-50 years at the level of very low physical activity-sedentary level;

¶ Acceptable macronutrient distribution ranges;

§Adequate intake;

** Dietary folate equivalent;

†† Estimated average requirement (EAR);

‡‡ Recommendation on saturated fat intake by Joint WHO/FAO;

¶¶ As retinol activity equivalents;

§§ As cholecalciferol. In the absence of adequate exposure to sunlight;

*** As alpha-tocopherol;

††† 1 kJ=0.24 kcal;

CHO=Carbohydrate;

DRI=Dietary reference intake;

FAO=Food and Agriculture Organizations;

NA=Not applicable;

RAE=Retinol activity equivalent;

SD=Standard deviation;

WHO=World Health Organization

**Table 3.** Percentage of Inuit women aged 19-44 years (n=106) below DRI‡‡

Nutrient	% of women below DRI	DRI
Calcium (mg)	35.8	1000*
Dietary fibre (g)	90.6	25*
Total folate†(μg)	28.3	320‡
Magnesium (mg)	17.0	255/265‡, ¶
Potassium (g)	62.3	4.7*
Vitamin A (RAE§) (μg)	16.0	500‡
Vitamin D** (μg)	60.3	200*
Vitamin E†† (mg)	99.1	12‡
Vitamin K (μg)	46.2	90*

* Adequate intake used for comparison;

† Dietary folate equivalent;

‡ Estimated average requirement used for comparison;

¶ 255 mg/d for women aged 19-30 years, 265 mg/d for women aged 31-44 years;

§Retinol activity equivalent;

** As cholecalciferol in the absence of adequate exposure to sunlight;

†† As alpha-tocopherol;

‡‡ EAR or AI used as cutoff value;

AI=Adequate intake;

DRI=Dietary reference intake;

EAR=Estimated average requirement;

RAE=Retinol activity equivalent

In the present study, inadequacy of vitamin A was moderately prevalent (16%) and lower than observed in a study (20). The latter study found that 60% of Inuit women in Nunavut aged 19-50 years had inadequate intake of vitamin A. Other studies have measured suboptimal vitamin A status in pregnant women and neonates in northern Canada (61,62). Thus, this topic warrants further investigation, particularly regarding possible seasonal variation in intake because of the possible role of vitamin A deficiency in infant congenital heart malformations (38).

The study women consumed excessive energy (5,657 kJ or 1,352 kcal higher than the minimum recommendation), which is likely associated with the high prevalence of overweight and obesity. Rates of obesity observed in this population compare with those previously observed in First Nation women (40.4% and 48.1%), which suggest that these patterns affect various Aboriginal populations in Canada (63). It is widely accepted that obesity is linked to the development of diabetes and cardiovascular diseases (64). Obesity and diabetes can also increase the risk of poor pregnancy outcomes (25-34) and may also predict an offspring's risk of development of chronic diseases later in life (39-41). In Canada, the financial burden of chronic diseases is significant, and the healthcare system is continuously challenged by the cost of service-delivery to remote communities. Investing in chronic disease-prevention programmes in these communities is essential to ade-quately manage healthcare costs and to maintain a high quality of life for northern populations. Since the health of women of childbearing age has a direct impact on the health of future generations, efforts that target this population group are of utmost importance.

The study women widely consumed store-bought foods, findings that further evidence the nutrition transition underway in Inuit populations (4,6,7). Non-nutrient-dense, store-bought foods were main contributors to energy, fat, carbohydrate and sugar intakes. Consumption of nutrient-rich fruits and vegetables was very low, contributing to less than 5% of energy intake. White bread was a notable contributor to folate, iron, and calcium—a benefit of wheat flour fortification. However, this high glycaemic food may cause fluctuations in blood glucose and insulin levels which over time are associated with gain in weight and increased risk of heart disease and diabetes (65). Traditional foods accounted for only 21% of energy intake but more than 50% of protein and iron intakes, highlighting the importance of these foods in quality of diet. Further, traditional foods are often good sources of vitamin A and D, which as mentioned previously, are important in ensuring positive reproductive outcomes.

These findings suggest that, to reduce energy and increase micronutrient intake among Inuit women of childbearing age, efforts targeting the replacement of non-nutrient-dense foods in the diet with traditional foods, fruits, vegetables, and whole grains are needed. Maternal diet may have life-long consequences on an offspring's health and susceptibility to chronic diseases in adulthood. Thus, investing in the promotion of Inuit women's health and nutrition can be expected to have sustained benefits on the health of this population.

### Limitations

Since the study was conducted in only three Inuit communities of Nunavut, the results may not be generalizable to all Inuit communities. Also, the FFQs are generally considered less than optimal to use to estimate the quantitative parameters of a usual dietary intake of population. However, the QFFQ used in the present study showed high agreement with the repeated 24-hour recall method, and the estimates of the study population's intakes were, thus, considered reasonable and appropriate for determining the dietary adequacy. Further, the QFFQ was developed for the unique Inuit population and contained a culturally-specific food list that captured most foods consumed in these communities. Benefits of using the QFFQ include that it will be a useful tool to monitor how the nutrition transition affects this population and will also allow for evaluating the effects of an intervention to improve dietary intake.

**Table 4.** Top 10 food sources of energy, macronutrients, sugar, and fibre, and % of dietary contributions* among Inuit women aged 19-44 years (n=106)

Food	Energy	Food	Protein	Food	Fat
Non-nutrient-dense foods	30.8	Traditional land foods	37.7	Non-nutrient-dense foods	22.2
Traditional land foods	13.2	Traditional sea foods	20.0	Beef and pork	21.4
Beef and pork	10.3	Beef and pork	12.8	Dairy	11.6
Traditional sea foods	7.8	Dairy	6.9	Traditional land foods	9.7
White breads	7.6	Chicken/turkey	4.5	Traditional sea foods	9.3
Dairy	6.8	Non-nutrient-dense foods	4.0	Noodles	6.7
Noodles	6.1	Noodles	3.7	White breads	6.4
Fruits	3.0	White breads	3.2	Chicken/turkey	3.9
Chicken/turkey	2.4	Other starches	1.5	Other starches	2.8
Other starches	1.9	Vegetables	0.8	Nuts	2.3
Total	89.9		95.1		96.3
Food	Carbohydrate	Food	Sugar	Food	Fibre
Non-nutrient-dense foods	51.0	Non-nutrient-dense foods	76.1	Fruits	16.7
White breads	10.6	Fruits	9.3	Non-nutrient-dense foods	15.7
Noodles	7.0	Dairy	6.1	White breads	11.7
Fruits	6.5	Wheat breads	1.4	Vegetables	10.4
Dairy	3.7	Cereals	1.4	Noodles	7.9
Rice	3.5	Vegetables	1.3	Beef and pork	6.7
Cereals	3.2	White breads	1.1	Wheat breads	6.2
Beef and pork	2.5	Noodles	1.0	Traditional land foods	5.8
Vegetables	2.3	Beef and pork	1.0	Cereals	5.4
Traditional land foods	2.3	Traditional land foods	0.3	Other starches	3.2
Total	92.6		99.0		89.7

*For each food item, the corresponding column reflects the percentage of contribution of the food to the respective nutrient's daily intake

**Table 5.** Top 10 food sources of selected micronutrients and % of dietary contributions* among Inuit women aged 19-44 years (n=106)

Food	Calcium	Food	Iron	Food	Folate	Food	Vitamin D
Dairy	31.2	Traditional land foods	43.7	White breads	31.1	Traditional sea foods	35.4
White breads	24.2	White breads	9.4	Non-nutrient-dense foods	24.2	Dairy	34.3
Non-nutrient-dense foods	20.9	Traditional sea foods	8.6	Dairy	6.1	Beef and pork	11.0
Noodles	5.6	Non-nutrient-dense foods	7.8	Vegetables	5.7	Non-nutrient-dense foods	10.4
Traditional sea foods	2.7	Beef and pork	7.2	Noodles	4.8	Traditional land foods	5.4
Traditional land foods	2.5	Cereals	6.0	Traditional land foods	4.8	Chicken/turkey	1.2
Other starches	2.5	Noodles	5.0	Fruits	4.3	White breads	1.1
Beef and pork	2.1	Dairy	2.1	Cereals	4.3	Other starches	0.5
Fruits	1.6	Vegetables	1.8	Beef and pork	3.6	Potatoes	0.4
Vegetables	1.5	Wheat breads	1.4	Other starches	3.5		
Total	94.8		93.0		92.4		99.7

*For each food item, the corresponding column reflects the percentage of contribution of the food to the respective nutrient's daily intake

### Conclusions

The Inuit women of childbearing age in Nunavut, Canada, exhibited excessive energy intake, high BMI, and low intakes of several essential nutrients, all of which can impact reproductive outcomes and the health of the offspring. High consumption of non-nutrient-dense foods likely contributed to the poor quality of diet. Education and behaviour-change strategies that promote good nutrition and prevent gain in weight are needed in this vulnerable population sub-group.

## ACKNOWLEDGEMENTS

The project was supported by American Diabetes Association Clinical Research award (no. 1-08-CR-57), the Government of the Northwest Territories Department of Health and Social Services, Health Canada, the Public Health Agency of Canada, and the Canadian Public Health Association. The authors thank their community staff for their great support and work on this project. They also thank the communities for their enthusiastic participation.
